# Community Cardiovascular Disease Risk From Cross-Sectional General Practice Clinical Data: A Spatial Analysis

**DOI:** 10.5888/pcd12.140379

**Published:** 2015-02-26

**Authors:** Nasser Bagheri, Bridget Gilmour, Ian McRae, Paul Konings, Paresh Dawda, Peter Del Fante, Chris van Weel

**Affiliations:** Author Affiliations: Bridget Gilmour, Ian McRae, Paul Konings, Paresh Dawda, Australian Primary Health Care Research Institute, Australian National University, Australia; Peter Del Fante, Healthfirst Network, Adelaide, Australia; Chris van Weel, Australian Primary Health Care Research Institute, Australian National University, Australia, and Department of Primary and Community Care, Radboud University Medical Centre, Nijmegen, the Netherlands.

## Abstract

**Introduction:**

Cardiovascular disease (CVD) continues to be a leading cause of illness and death among adults worldwide. The objective of this study was to calculate a CVD risk score from general practice (GP) clinical records and assess spatial variations of CVD risk in communities.

**Methods:**

We used GP clinical data for 4,740 men and women aged 30 to 74 years with no history of CVD. A 10-year absolute CVD risk score was calculated based on the Framingham risk equation. The individual risk scores were aggregated within each Statistical Area Level One (SA1) to predict the level of CVD risk in that area. Finally, the pattern of CVD risk was visualized to highlight communities with high and low risk of CVD.

**Results:**

The overall 10-year risk of CVD in our sample population was 14.6% (95% confidence interval [CI], 14.3%–14.9%). Of the 4,740 patients in our study, 26.7% were at high risk, 29.8% were at moderate risk, and 43.5% were at low risk for CVD over 10 years. The proportion of patients at high risk for CVD was significantly higher in the communities of low socioeconomic status.

**Conclusion:**

This study illustrates methods to further explore prevalence, location, and correlates of CVD to identify communities of high levels of unmet need for cardiovascular care and to enable geographic targeting of effective interventions for enhancing early and timely detection and management of CVD in those communities.

## Introduction

Cardiovascular disease (CVD) is a leading cause of death and disease burden across the world, and the burden is expected to increase as the population ages (1–3). CVD is the most expensive disease in Australia; it accounted for $7.9 billion, or 11%, of health spending from 2009 to 2010 (1).

The most commonly used CVD risk prediction algorithms are those derived from the Framingham Risk Equation (FRE), which is used in general practice (GP) to assess risks for individual patients (4). The trend in primary prevention of CVD in GPs has been to move away from assessment of relative CVD risk factors toward assessment and management of these factors as absolute CVD risk (5,6).

Best-value prevention strategies require knowledge and contextualized understanding of people, communities, and environments, as well as variations in CVD risk. Although clinically proven tools are available for assessing risk factors in individuals, most at-risk individuals never take part in such assessment until disease progression is under way. Although imprecise proxies for risk can be used to make community-based risk estimates, there is still a considerable knowledge gap; no fine-grained population tools exist to directly predict “hotspots” for future CVD risk from GP clinical data.

Few studies have attempted to examine spatial variation of CVD risk at a smaller geographic scale across the world. Noble et al examined the feasibility of mapping chronic disease risk in general and created a small-area map of diabetes risk from GP clinical records in the United Kingdom (7). In Australia, Tideman et al compared the CVD risk of a population survey sample from northwest Adelaide with a nearby rural population but did not look at the variation within the survey population catchment (8). This is the first study that visualizes the pattern of CVD risk at a small-area scale from GP clinical records to explore possible clusters or hotspots of CVD risk in communities. The small area used, Statistical Area Level 1 (SA1), has a population size between 200 to 800 people, which is approximately equal to the size of a US census block (315 people on average) (9).

The main objective of this study was to explore patterns of CVD risk in people across small areas and investigate the association between area-level socioeconomic status and CVD risk patterns. This approach allows the production of fine-grained maps of CVD risk for use by clinicians and policy makers to enable geographic targeting of interventions in communities.

## Methods

### Data sources

De-identified clinical practice data from 2010 through 2012 were drawn from a large, multisite GP in northwest Adelaide in South Australia at the end of 2012. The data were linked to SA1s using methods described by Mazumdar et al in 2014 (10). There are 148 SA1s in the study area, and 14 other general practices operate in the same area. Although patients came from a wide area, they were concentrated on the LeFevre Peninsula and comprised 18% of the population of this area. Overall, data on 14,969 active patients were extracted from the practice; 8,630 of these patients were aged 30 to 74 years with no prior history of CVD (ie, stroke, chronic heart disease, peripheral vascular disease, or heart failure). 

Patients were excluded if data were not available for 7 risk factors for CVD, which are required to calculate the FRE. A major part of the analysis was based on geography, so the patients were classified according to the SA1 in which they resided. The patients from the clinic mostly resided on the LeFevre Peninsula and in surrounding areas, but some came from farther distances. To maintain confidentiality, the study excluded those SA1s that contained fewer than 5 patients (701 patients in total), leaving a sample of 4,740 patients. Under Australian health care, patients access primary health care as the point of entry into the health care system, and each year approximately 85% of the population has contact with a GP. Therefore, to the degree that GPs choose to participate in studies such as this one, it is possible to obtain high patient coverage.

### Characteristics associated with CVD risk and identifying areas of high risk

We used the FRE to evaluate 5-year and 10-year absolute risks of CVD for individuals based on GP clinical data for the northwest Adelaide area. The FRE, a risk model designed for use on individuals’ clinical data, is well-suited for producing population-level risk estimates (11,12). The FRE accounts for age, sex, total cholesterol, high-density lipoprotein cholesterol, systolic blood pressure, smoking status, and whether a patient has diabetes to estimate the patient’s risk for developing CVD in the next 10 years. The recommended scoring system has been in use since 1991 (13). We also calculated patients’ 5-year risk of CVD using the FRE and compared these data with the 10-year data to assess patterns. 

The National Vascular Disease Prevention Alliance in Australia defined CVD risk category on the basis of FRE as follows: an absolute risk of CVD events over 10 years higher than 20% is high risk, from 10% to 20% is moderate risk, and less than 10% is low risk. Body mass index (BMI, kg/m^2^) was not used in the FRE, but because it is a major risk factor for CVD we included the distribution of CVD risk by BMI category. We used World Health Organization recommendations for BMI cut-offs as follows: underweight, less than 18.5; normal weight, 18.5 to 24.9; overweight 25.0 to 29.9; and obese, 30.0 or higher.

First, we linked the de-identified patient records, including calculated absolute 5-year and 10-year CVD risk, to the corresponding SA1 within the practice catchment. Second, the individual risk scores were aggregated to SA1 level by calculating mean risk for each SA1. Third, the area level of CVD risk was visualized to examine the areas of high and low probability of developing CVD risk over the next 10 years in the study area.

An index of relative socioeconomic disadvantage (IRSD) developed by the Australian Bureau of Statistics was linked to the corresponding SA1 to make comparisons with the pattern of absolute CVD risk (14,15). IRSD is a general socioeconomic index derived from census variables related to disadvantage, such as low income, low educational attainment, unemployment, and dwellings without motor vehicles. We ran a linear regression model to investigate the relationship between CVD risk and ISRD, adjusting for demographic variables.

To identify areas with high risk (hotspots) and low risk (coldspots) for CVD, a continuous heat map of CVD risk was generated using the inverse distance weighting (IDW) technique. The IDW technique generates an interpolated and smoothed risk surface, with an anticipated statistical resolution at neighborhood (or community) level to accurately identify high-risk areas (16). Cluster and outlier analysis with Anselin local Moran’s I and Getis-Ord G techniques were used to investigate potential clusters of hotspots in the study areas (Appendix). We compared IDW with Natural Neighbor and kriging smoothing methods (17). However, the output pattern for these 2 techniques was the same. The parameters included to generate a smoothed pattern in the IDW method include Manhattan distance as distance parameter, number of units (12 points), and centroids of each SA1. The tertile of the index of relative socioeconomic disadvantage was mapped for each SA1 to compare with the pattern of CVD risk at the SA1 level in the study area (11).

We used Stata version 12.1 (StataCorp, LP) to calculate the CVD risk scores and conduct descriptive analyses for our sample population and ArcGIS version 10.2 (Esri) to conduct spatial analyses and mapping. The spatial analysis was restricted to the LeFevre Peninsula area of the GP catchment, where there was the highest penetration of the practice; therefore, the mapped data are in an area where the numbers of patients are larger and estimated risks more stable. The study obtained ethics approval from the Australian National University human ethics committee (protocol 2014/174).

## Results

### Patient characteristics

Overall, patients who were male, smokers, overweight or obese, living in low socioeconomic areas (Table), and who had high blood pressure and total cholesterol had higher prevalence of CVD risk over 5 or 10 years than did their counterparts. The overall 5- and 10-year CVD risk in people aged 30 to 74 years at the GP level was 6.8% (95% CI, 6.6%–7.0%) and 14.6% (95% CI, 14.3%–14.9%), respectively. The percentage of sample population with high risk was 26.6%, moderate risk was 30.0%, and low risk was 43.6%. Both 5-year and 10-year CVD risk rose as BMI values increased.

**Table:** Cohort (N = 4,740) Characteristics and 5-Year and 10-Year Cardiovascular Disease (CVD) Risk at General Practice Level, Adelaide, Australia, 2012

Characteristic	No. (%)	5-Year CVD Risk, %	10-Year CVD Risk, %
**Age^a^, y**			
30–34	179 (3.8)	0.6	1.8
35–39	287 (6.0)	1.3	3.6
40–44	441 (9.3)	2.6	6.5
45–49	579 (12.2)	3.8	9.1
50–54	807 (17.0)	5.4	12.3
55–59	779 (16.4)	7.3	15.8
60–64	761 (16.0)	9.6	19.9
65–69	502 (10.6)	11.0	22.3
70–74	405 (8.5)	14.1	27.2
**Sex^a^**			
Male	1,990 (42.0)	9.9	20.2
Female	2,750 (58.0)	4.6	10.5
**Body mass index category^b^ (kg/m^2^)**			
Underweight (<18.5)	14 (0.6)	2.3	5.5
Normal (18.5–24.9)	399 (18.4)	5.2	11.4
Overweight (25.0–29.9)	723 (33.3)	7.0	14.9
Obese (≥30.0)	1,036 (47.7)	8.6	17.6
**Smoking status^a^**			
Smoker	2,206 (46.5)	9.5	19.5
Nonsmoker	2,534 (53.5)	4.5	10.3
**Index of relative socioeconomic disadvantage**			
Most disadvantaged areas	1,461 (30.8)	7.9	16.4
Moderately disadvantaged areas	1,604 (33.8)	6.6	14.2
Least disadvantaged areas	1,675 (35.3)	6.2	13.4
**Total**	4,740 (100.0)	6.8 **c**	14.6^d^

a Risk factor was included in the Framingham equation.

b Data were available for body mass index for only 45.8% of the sample, so the total number of patients in this category does not sum to 4,740.

c 95% Confidence interval, 6.6%–7.0%.

d 95% Confidence interval, 14.3%–14.9%.

### Spatial pattern of CVD risk and socioeconomic status

**Figure 1:**
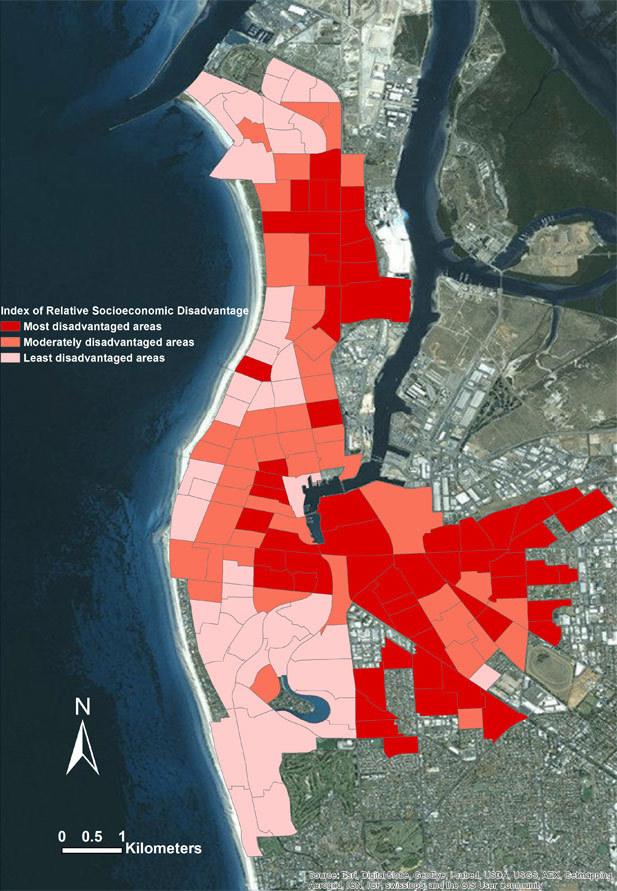
Pattern of an index of relative socioeconomic disadvantage in study area, Adelaide, Australia, 2012.

The most disadvantaged population lives in the eastern part of the study area near the industrialized regions, and the least disadvantaged population lives in the western beach areas (Figure 1). The overall pattern of 10-year risk of CVD varied across the SA1s (range, 6%–28%). The 10-year CVD risk was significantly higher in the most disadvantaged areas (16.4%) than the least disadvantaged areas (13.4%) (Table ).

**Figure 2:**
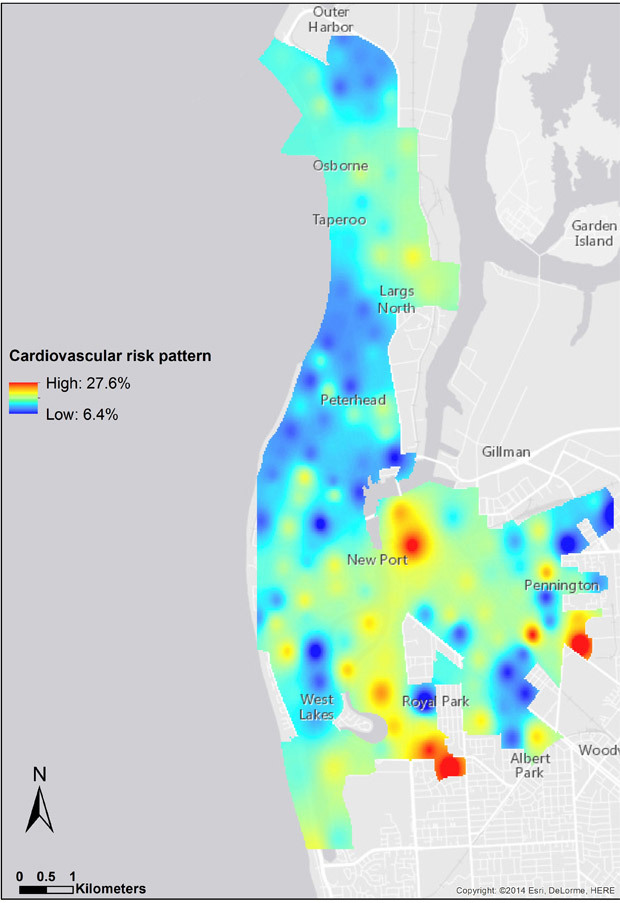
Smoothed pattern of cardiovascular disease risk in the study area, Adelaide, Australia, 2012.

The interpolated surface of the 10-year CVD risk shows clusters (risk of CVD) across the study area (Figure 2). The hotspots are seen in the most disadvantaged areas in the eastern and central parts of the LeFevre Peninsula. Clusters with high CVD risk values were significantly different from neighboring clusters’ CVD risk values (*z* score, 2.16; *P* = .03).

The number of active patients varied from 5 to 151 (median,16) across the SA1s in the study area. However, very few SA1s were small; only 4% of SA1s with high CVD risk had greater than 5 and fewer than 10 patients. This rate was 6% for low CVD risk areas.

Statistical and visual comparison of the spatial pattern of CVD risk with the IRSD pattern indicates that the risk of CVD is highest in the most disadvantaged areas, consistent with the results shown in Table 1 and Figure 2. Furthermore, the regression model results showed a significant negative relationship between CVD risk and IRSD (*P* < .001), which means CVD risk is more prevalent in disadvantaged areas.

## Discussion

This research aimed to identify area-level CVD risk and the proportion of the population at high risk using GP clinical records. Patients’ 5- and 10-year risk scores were generated on the basis of the Framingham risk prediction model, and these estimated scores were aggregated and visualized at the SA1 level. Finally a “heat map” interpolation surface of CVD risk was created to highlight the hotspots (high-risk areas) and coldspots (low-risk areas) in the study area. To our knowledge this is the first time area-level CVD risk, hotspots, and clustering in CVD risk have been studied using de-identified GP clinical records. We found that the proportion of patients at high risk for CVD risk was significantly higher in the communities of low socioeconomic status than in those of high socioeconomic status.

A considerable amount of literature has been published on the validity and generalizability of the FRE, which has been recommended as the most reliable method of predicting CVD risk in the United States (18–20). Many studies have demonstrated that the Framingham method of predicting risk is accurate when used on other populations, including most Australians and Dutch people (21–23). However, it is not generalizable to every population, and it can significantly underestimate the CVD risk of Aborigines (24). The Aboriginal data were poorly recorded in our GP dataset, so we were not able to evaluate a CVD risk pattern in Aboriginal people. However, the proportion of Aboriginal and Torres Strait Islander people in the general population in our study area was approximately 2%. Regardless of its limitations, the FRE is recommended by the National Heart Foundation of Australia to calculate CVD risk of Australians (25).

Of 4,740 patients aged 30 or older, 1,260 (26.6%) had a 10-year CVD risk score of greater than 20%. This proportion is higher than expected (16%–20%) (26). However, this finding may have been related to the fact that most patients in the sample were overweight or obese (81%), older (68% older than 50 years), and diabetic (10.1%). As the number of patients in each SA1 varied, and in some cases was quite small, CVD risk varied not only due to “real” effects but also to sample size variability. We expected to overestimate the overall CVD risk, in particular, for the younger age groups. For example, of 716 active patients in the age range 30 to 34 years, we had data for all the risk factors on only 179 patients. Doctors may not have considered the other 537 patients to be at high risk of CVD and thus did not feel it was necessary to measure, for example, their cholesterol levels. However, the 179 patients for whom who we did have a complete data set were more likely to have obvious risk factors, such as obesity, which would have compelled their doctors to make all the measurements with which to calculate CVD risk. Therefore, the patients whose data we were missing may be at lower risk for CVD; consequently, we may have overestimated CVD risk for this population, and analyses on a larger sample are recommended. 

Furthermore, we looked at SA1 level CVD risk patterns for patients who were older than 70 years. The CVD risk maps were flattened for 78% of SA1s with a CVD risk of greater than 20% (high risk) in the study area. We compared the age and sex pattern of the analysis sample (4,740) with the entire original cohort of 14,969 and population aged 30 to 74 years (38,770) for the geographic area of interest (Appendix). The analysis sample comprises an older population, but the original cohort is much the same as the overall population. For example, the percentages of population older than 40 years are 90% in the analysis sample, 86% in the original cohort, and 78% in the area of geographic interest. The greater CVD risk in older patients is unsurprising, because this group usually has higher prevalence of CVD risk factors compared with other adults. Similar findings were seen among American adults aged 20 years or older and Canadian adults aged 30 to 74 years who participated in a cross-sectional sample of 5,440 and 1,293, respectively (27,28).

This research addressed a significant public health problem, that of identifying the spatial distribution of CVD risk in Australia in a timely way so that prevention services can be more efficiently distributed. This project also allows risk profiles to be considered in relation to socioeconomic characteristics of areas. Consequently, our approach may be useful in describing and exploring spatial inequalities in the distribution of CVD risk, or any chronic disease for which risk modeling is available, that contributes to our understanding of health inequalities.

The development of a tool for monitoring disease risk has the potential to improve service delivery, policy development, research, and ultimately health outcomes, which would be particularly beneficial for people living in underserviced areas. No tools exist in Australia to predict risk hotspots in a timely manner, which means that development of chronic disease prevention policies at the national, state, and local levels are not informed by the most current information about disease risk in specific populations. Additionally, this method enables ecological studies of relationship between the area risk and socioeconomic status and built environment characteristics such as access to green spaces and fast-food outlets. This method can be used to estimate prevalence of CVD risk at different geographical scales from GP catchments to the national level, using demographic and clinical risk factors.

A limitation of this study is that the FRE may underestimate risk for people who take lipid-lowering or antihypertensive medication or people who have recently stopped smoking (29). However, current clinical practice is to calculate people’s risk even if they are taking medications (30).

The spatial analysis indicated that high CVD risk clusters were located in the most disadvantaged areas. This finding suggests that public health policy makers should focus on these hotspots and plan for active case finding (eg, screening tests) to operate early preventive interventions to reduce population CVD risk in those areas.

This approach provides an opportunity for researchers who have access to GP-based clinical data to further explore prevalence, location, and correlates of CVD and is applicable anywhere that these data are available. This method can be used as a tool to identify areas of high levels of unmet need for cardiovascular care, which could enable geographic targeting of effective interventions for enhancing early and timely detection and management of CVD in those communities. Furthermore, this study demonstrates that GP data can help identify public health priorities.

## Appendix.

Clusters of hotspots in the study areas, Adelaide, Australia, 2012. [This file is available for download as a Microsoft Word document.]
