# Supplementary material for: Community Cardiovascular Disease Risk From Cross-Sectional General Practice Clinical Data: A Spatial Analysis
Source: Prev Chronic Dis. 2015 Feb 26;12:E26. doi: 10.5888/pcd12.140379 (PMC4344355; doi:10.5888/pcd12.140379)
Supplement: Supplementary file 1 [file 14_0379_01.doc]

**Spatial Autocorrelation Report (Moran’s I)**


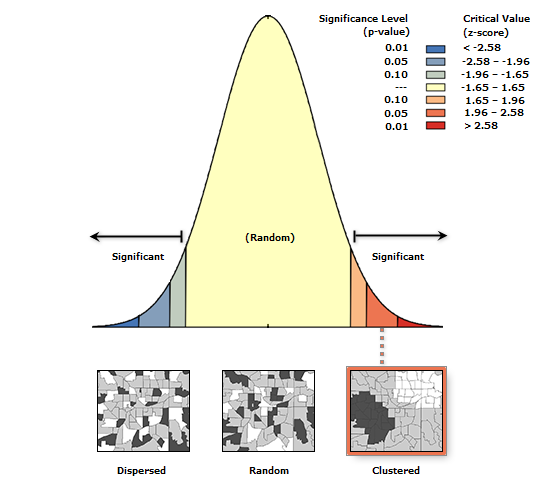


| **Global Moran's I Summary** | |
| --- | --- |
| **Moran's Index:** | 0.08 |
| **Expected Index:** | -0.006 |
| **Variance:** | 0.001 |
| **z-score:** | 2.16 |
| **p-value:** | 0.03 |

| **Dataset Information** | |
| --- | --- |
| **Input Feature Class:** | SA1_2011_AUST_Points |
| **Input Field:** | CVD_RISK10 |
| **Conceptualization:** | INVERSE_DISTANCE |
| **Distance Method:** | MANHATTAN |
| **Row Standardization:** | False |
| **Distance Threshold:** | 0.009 Degrees |
| **Weights Matrix File:** | None |
| **Selection Set:** | False |

**Table 1.supplemntal for exclusion criteria**

| **Exclusion criteria** | **Running total** |
| --- | --- |
| Original active patient | 14,969 |
| Age <30 (4,641) or >74 (1,465) = 6,106 | 14,969 – 6,106 = 8,863 |
| CVD = 233 | 8,863 – 233 = 8,630 |
| No SA1 data (not geocoded) = 254 | 8,630 – 254 = 8,376 |
| Missing variables (sex 38, TC 1105, HDL 2,348, smoking 1,051, systolic BP 883) = 2,935  (the individual variables don’t add up to the total number of people with missing variables because some people are missing more than one variable) | 8,376 – 2,935 = 5,441 |
| SA1 contains <5 people = 701 | 5,441 – 701 = 4,740 |
| Final dataset | 4,740 |

**Table 2: Association between IRSD and CVD risk adjusted for sex and age**

|  | **Coefficient** | **Std. Err.** | **t** | **P>t** | **95% CI** |
| --- | --- | --- | --- | --- | --- |
| **IRSD** | -0.01 | 0.00 | -9.7 | 0.001 | -0.01, -0.00 |
| **Age group (reference 30-34)** | | | | | |
| 35-39 | 2.1 | .73 | 2.9 | 0.001 | 0.7, 3.6 |
| 40-44 | 4.3 | .68 | 6.3 | 0.001 | 2.9, 5.6 |
| 45-49 | 7.1 | .66 | 10.7 | 0.001 | 5.8, 8.4 |
| 50-54 | 10.0 | .64 | 15.7 | 0.001 | 8.8, 11.3 |
| 55-59 | 13.3 | .64 | 20.8 | 0.001 | 12.1, 14.6 |
| 60-64 | 17.1 | .64 | 0.6 | 0.001 | 15.8, 18.4 |
| 65-69 | 20.0 | .67 | 29.7 | 0.001 | 18.7, 21.4 |
| 70-74 | 24.8 | .69 | 35.6 | 0.001 | 23.4, 26.1 |
| **Sex (reference male)** | | | | | |
| Female | -9.1 | .22 | -40.0 | 0.001 | -9.6, -8.7 |
| _cons | 17.8 | 1.1 | 14.9 | 0.001 | 15.5, 20.2 |

**Table 3:** Comparison of analysis sample, original cohort with demographics of study area

|  | Analysis sample (4740)-% | Original cohort (8863)-% | | Total population of study area (38770)-% |
| --- | --- | --- | --- | --- |
| Age |  | | | |
| 30-39 | 9.7 | 19.8 | | 22.0 |
| 40-49 | 21.5 | 21.9 | | 25.0 |
| 50-59 | 33.4 | 29.6 | | 25.7 |
| 60-69 | 26.6 | 21.0 | | 19.9 |
| 70-74 | 8.5 | 7.7 | | 7.4 |
| **Sex** |  | | | |
| Male | 42 | | 44 | 49 |
| Female | 58 | | 55 | 51 |

Table 4: Data points used for creating maps:

| Statistical area (SA1) codes | Index of relative socioeconomic disadvantage (tertile) | Active patient per SA1 | 10-year cardiovascular risk (%) | High risk patients (%) |
| --- | --- | --- | --- | --- |
| 40401109007 | 3 | 6 | 17.3 | 50.0 |
| 40401109018 | 2 | 5 | 7.8 | 5.9 |
| 40401109105 | 3 | 5 | 16.2 | 20.0 |
| 40401109122 | 1 | 5 | 20.4 | 60.0 |
| 40401109206 | 3 | 5 | 9.0 | 5.9 |
| 40401109213 | 3 | 5 | 12.7 | 5.9 |
| 40401109215 | 2 | 6 | 5.7 | 5.9 |
| 40401109323 | 2 | 5 | 6.8 | 5.9 |
| 40401109401 | 1 | 9 | 14.8 | 33.3 |
| 40401109402 | 1 | 5 | 6.4 | 5.9 |
| 40401109403 | 1 | 11 | 19.0 | 36.4 |
| 40401109406 | 1 | 6 | 24.2 | 50.0 |
| 40401109407 | 1 | 13 | 16.2 | 30.8 |
| 40401109408 | 1 | 6 | 19.5 | 33.3 |
| 40401109411 | 1 | 10 | 12.5 | 20.0 |
| 40401109412 | 1 | 8 | 20.0 | 37.5 |
| 40401109413 | 2 | 7 | 10.7 | 42.9 |
| 40401109503 | 3 | 12 | 15.2 | 25.0 |
| 40401109504 | 1 | 5 | 18.0 | 20.0 |
| 40401109505 | 1 | 5 | 13.5 | 40.0 |
| 40401109506 | 1 | 7 | 13.8 | 14.3 |
| 40401109510 | 1 | 6 | 26.3 | 66.7 |
| 40401109511 | 1 | 9 | 16.3 | 44.4 |
| 40401109512 | 1 | 5 | 7.1 | 5.9 |
| 40401109516 | 2 | 6 | 17.2 | 33.3 |
| 40401109531 | 3 | 9 | 15.3 | 33.3 |
| 40401109601 | 2 | 16 | 16.6 | 50.0 |
| 40401109602 | 3 | 25 | 16.5 | 36.0 |
| 40401109603 | 1 | 9 | 18.0 | 44.4 |
| 40401109604 | 2 | 21 | 15.0 | 23.8 |
| 40401109605 | 3 | 7 | 18.6 | 42.9 |
| 40401109606 | 3 | 8 | 15.0 | 37.5 |
| 40401109607 | 3 | 6 | 12.7 | 33.3 |
| 40401109608 | 2 | 7 | 13.6 | 28.6 |
| 40401109609 | 2 | 17 | 12.4 | 17.6 |
| 40401109610 | 3 | 12 | 20.3 | 33.3 |
| 40401109611 | 3 | 6 | 18.9 | 50.0 |
| 40401109612 | 2 | 18 | 16.1 | 33.3 |
| 40401109613 | 1 | 16 | 17.0 | 31.3 |
| 40401109614 | 2 | 17 | 18.1 | 29.4 |
| 40401109615 | 3 | 9 | 18.6 | 22.2 |
| 40401109616 | 3 | 9 | 20.4 | 66.7 |
| 40401109617 | 3 | 8 | 15.5 | 37.5 |
| 40401109618 | 3 | 12 | 18.0 | 41.7 |
| 40401109619 | 3 | 9 | 7.8 | 11.1 |
| 40401109620 | 3 | 9 | 20.0 | 44.4 |
| 40401109621 | 3 | 22 | 19.9 | 45.5 |
| 40401109622 | 3 | 9 | 14.8 | 22.2 |
| 40401109624 | 3 | 12 | 15.1 | 33.3 |
| 40401109625 | 3 | 7 | 18.6 | 28.6 |
| 40401109626 | 3 | 15 | 14.7 | 26.7 |
| 40401109627 | 3 | 14 | 10.6 | 14.3 |
| 40401109629 | 3 | 8 | 21.8 | 50.0 |
| 40401109630 | 3 | 5 | 12.4 | 20.0 |
| 40401109631 | 3 | 12 | 15.1 | 41.7 |
| 40401109632 | 2 | 16 | 13.8 | 25.0 |
| 40401109633 | 1 | 7 | 15.4 | 28.6 |
| 40401109634 | 1 | 23 | 18.8 | 34.8 |
| 40401109703 | 1 | 5 | 27.7 | 40.0 |
| 40401109704 | 2 | 7 | 12.1 | 28.6 |
| 40401109705 | 1 | 10 | 9.9 | 20.0 |
| 40401109706 | 1 | 7 | 20.5 | 42.9 |
| 40401109708 | 1 | 5 | 12.0 | 5.9 |
| 40401109709 | 1 | 9 | 16.3 | 33.3 |
| 40401109715 | 2 | 9 | 16.3 | 33.3 |
| 40401109716 | 1 | 9 | 24.4 | 44.4 |
| 40401109717 | 2 | 8 | 9.5 | 12.5 |
| 40401109734 | 2 | 28 | 11.1 | 17.9 |
| 40401109735 | 3 | 9 | 9.5 | 11.1 |
| 40402109901 | 3 | 7 | 10.0 | 14.3 |
| 40402109902 | 1 | 21 | 15.2 | 28.6 |
| 40402109903 | 3 | 47 | 10.4 | 21.3 |
| 40402109904 | 3 | 41 | 14.3 | 26.8 |
| 40402109905 | 2 | 49 | 11.9 | 12.2 |
| 40402109906 | 3 | 26 | 16.5 | 34.6 |
| 40402109907 | 2 | 33 | 12.2 | 18.2 |
| 40402109908 | 2 | 22 | 10.6 | 18.2 |
| 40402109909 | 2 | 76 | 14.0 | 22.4 |
| 40402109910 | 3 | 58 | 11.9 | 19.0 |
| 40402109911 | 3 | 62 | 10.3 | 16.1 |
| 40402109912 | 2 | 77 | 13.6 | 19.5 |
| 40402109913 | 1 | 49 | 17.2 | 34.7 |
| 40402109914 | 2 | 51 | 15.8 | 31.4 |
| 40402109915 | 2 | 44 | 13.6 | 18.2 |
| 40402109916 | 2 | 22 | 13.5 | 27.3 |
| 40402109917 | 1 | 17 | 8.2 | 5.9 |
| 40402109918 | 2 | 23 | 17.3 | 43.5 |
| 40402109919 | 3 | 36 | 10.8 | 13.9 |
| 40402109920 | 2 | 39 | 16.3 | 38.5 |
| 40402109921 | 2 | 29 | 9.2 | 10.3 |
| 40402109923 | 2 | 17 | 12.0 | 17.6 |
| 40402109924 | 1 | 14 | 16.5 | 35.7 |
| 40402109925 | 2 | 20 | 14.3 | 25.0 |
| 40402109926 | 2 | 31 | 11.3 | 9.7 |
| 40402109927 | 1 | 17 | 13.4 | 35.3 |
| 40402109928 | 2 | 51 | 13.6 | 29.4 |
| 40402109929 | 1 | 20 | 12.0 | 20.0 |
| 40402109930 | 3 | 14 | 13.7 | 21.4 |
| 40402109931 | 3 | 26 | 11.7 | 11.5 |
| 40402109932 | 2 | 15 | 16.6 | 26.7 |
| 40402109933 | 2 | 32 | 18.9 | 34.4 |
| 40402109934 | 1 | 18 | 15.1 | 27.8 |
| 40402109935 | 2 | 19 | 13.9 | 26.3 |
| 40402109937 | 3 | 35 | 10.7 | 17.1 |
| 40402110001 | 1 | 47 | 16.2 | 27.7 |
| 40402110002 | 1 | 64 | 17.3 | 26.6 |
| 40402110003 | 3 | 86 | 13.6 | 23.3 |
| 40402110004 | 1 | 79 | 15.1 | 26.6 |
| 40402110005 | 3 | 115 | 15.0 | 27.0 |
| 40402110006 | 3 | 57 | 11.6 | 19.3 |
| 40402110007 | 3 | 69 | 11.8 | 21.7 |
| 40402110008 | 2 | 69 | 11.9 | 13.0 |
| 40402110009 | 2 | 54 | 11.3 | 16.7 |
| 40402110010 | 3 | 73 | 11.8 | 19.2 |
| 40402110011 | 2 | 75 | 14.8 | 25.3 |
| 40402110012 | 2 | 40 | 11.6 | 20.0 |
| 40402110013 | 3 | 98 | 12.1 | 19.4 |
| 40402110014 | 3 | 48 | 12.2 | 16.7 |
| 40402110015 | 3 | 111 | 11.1 | 13.5 |
| 40402110016 | 2 | 45 | 16.9 | 35.6 |
| 40402110017 | 1 | 40 | 16.1 | 32.5 |
| 40402110018 | 1 | 43 | 16.4 | 30.2 |
| 40402110019 | 3 | 66 | 13.2 | 16.7 |
| 40402110020 | 1 | 81 | 19.1 | 43.2 |
| 40402110021 | 3 | 106 | 14.3 | 26.4 |
| 40402110022 | 1 | 10 | 18.8 | 50.0 |
| 40402110023 | 1 | 78 | 16.3 | 32.1 |
| 40402110024 | 3 | 121 | 14.9 | 28.1 |
| 40402110025 | 2 | 64 | 13.9 | 26.6 |
| 40402110026 | 1 | 88 | 17.3 | 36.4 |
| 40402110027 | 1 | 109 | 17.5 | 32.1 |
| 40402110029 | 1 | 62 | 14.5 | 27.4 |
| 40402110030 | 1 | 71 | 14.1 | 26.8 |
| 40402110031 | 2 | 60 | 14.9 | 25.0 |
| 40402110032 | 1 | 48 | 15.1 | 27.1 |
| 40402110033 | 2 | 151 | 15.7 | 31.1 |
| 40402110034 | 1 | 49 | 15.6 | 28.6 |
| 40402110035 | 2 | 56 | 16.4 | 30.4 |
| 40402110036 | 2 | 80 | 15.1 | 30.0 |
| 40402110037 | 3 | 51 | 15.6 | 31.4 |
| 40402110038 | 3 | 47 | 15.0 | 27.7 |
| 40402110101 | 1 | 6 | 14.6 | 16.7 |
| 40402110102 | 1 | 18 | 14.1 | 33.3 |
| 40402110104 | 1 | 5 | 7.4 | 5.9 |
| 40402110105 | 1 | 7 | 13.9 | 28.6 |
| 40402110106 | 1 | 15 | 25.1 | 80.0 |
| 40402110107 | 1 | 31 | 21.2 | 41.9 |
| 40402110108 | 1 | 9 | 7.3 | 5.9 |
| 40402110110 | 1 | 9 | 18.3 | 33.3 |
| 40402110111 | 1 | 13 | 21.2 | 46.2 |
| 40402110112 | 1 | 7 | 17.6 | 28.6 |
| 40402110113 | 1 | 11 | 10.6 | 18.2 |
| 40402110114 | 1 | 14 | 16.9 | 35.7 |
| 40402110115 | 1 | 7 | 12.6 | 14.3 |
| 40402110116 | 1 | 8 | 18.2 | 25.0 |
| 40402110117 | 2 | 16 | 17.9 | 37.5 |
| 40402110118 | 1 | 19 | 18.4 | 36.8 |
| 40402110119 | 1 | 5 | 16.6 | 40.0 |
| 40402110120 | 1 | 16 | 12.6 | 31.3 |
| 40402110121 | 1 | 12 | 16.8 | 33.3 |
| 40402110123 | 1 | 9 | 15.6 | 22.2 |
| 40402110124 | 1 | 5 | 18.1 | 40.0 |
| 40402110125 | 2 | 19 | 17.3 | 36.8 |
| 40402110126 | 2 | 10 | 16.7 | 40.0 |
| 40402110127 | 2 | 22 | 14.3 | 22.7 |
